# Tracking Changes in Community Liveability, Interpersonal Connection and Loneliness Among Older Adults in Taiwan

**DOI:** 10.1093/geroni/igaf122.3291

**Published:** 2025-12-31

**Authors:** Shiau-Fang Chao, Jun-Rong Huang, Tzai-Hung Wen, Hsuanlei Shao

**Affiliations:** National Taiwan University, Taipei, Taipei, Taiwan (Republic of China); National Taiwan University, Taipei, Taipei, Taiwan (Republic of China); National Taiwan University, Taipei, Taipei, Taiwan (Republic of China); Taipei Medical University, Taipei City, Taipei, Taiwan (Republic of China)

## Abstract

**Background:**

Loneliness has high prevalence among older adults, yet our understanding of how community liveability affects this condition remains limited. Community liveability represents a modifiable environmental factor that may protect against loneliness. Particularly scarce are longitudinal studies examining how changes in environmental and social factors influence loneliness, especially in Asian contexts like Taiwan where rapid aging and changing family structures make this research critically important.

**Methods:**

This study employed multilevel modeling to analyze data from 1,834 older adults (3,668 observations) drawn from the 2015 and 2019 waves of the Taiwan Longitudinal Study on Aging.

**Findings:**

1. At the within-person level, increases in individual housing satisfaction and medical treatment accessibility over time were significantly associated with decreased feelings of loneliness. Additionally, maintaining connections with siblings and relatives over time effectively reduced individuals’ experiences of loneliness. 2. At the between-person level, all four community liveability factors, including housing satisfaction, medical treatment accessibility, perceived neighborhood safety, and environmental satisfaction, significantly predicted lower levels of loneliness. 3. Furthermore, at the between-person level, individuals who maintained connections with siblings, relatives, neighbors, or friends reported lower levels of loneliness compared to those lacking interpersonal connections.

**Conclusions and Implications:**

Both community liveability factors and interpersonal connections serve as protective factors against loneliness among older adults. The longitudinal findings emphasize that sustained improvements in environmental conditions and maintaining consistent social connections over time are particularly effective in reducing loneliness.

